# Vulvar “proximal-type” epithelioid sarcoma: report of a case and review of the literature

**DOI:** 10.1186/1746-1596-8-122

**Published:** 2013-07-25

**Authors:** Lodovico Patrizi, Giacomo Corrado, Maria Saltari, Letizia Perracchio, Chiara Scelzo, Emilio Piccione, Enrico Vizza

**Affiliations:** 1 Department of Oncological Surgery, Gynecologic Oncology Unit, “Regina Elena” National Cancer Institute, Via Elio Chianesi 53, Rome, 00144, Italy; 2 Surgery Department, Gynecology Section and Obstetrics, Tor Vergata University, Rome, Italy; 3 Pathology Department, “Regina Elena” National Cancer Institute, Rome, Italy; 4 Surgery Department, Gynecologic Oncology Unit, “Regina Elena” National Cancer Institute, Rome, Italy

**Keywords:** Vulvar “proximal-type” epithelioid sarcoma, Radical vulvectomy, Vulvar cancer

## Abstract

**Background:**

The “proximal-type” epithelioid sarcoma is a very rare kind of mesenchimal tumor characterized by the difficulty in histological diagnosis and the very aggressive biological behavior.

**Case:**

We report of a case of a 63 years old woman with a vulvar “proximal-type” epithelioid sarcoma that underwent a radical surgical staging followed by an adjuvant radiotherapy. She is on follow-up care for 14 months and there is no clinical evidence of disease.

**Conclusion:**

Even if quite rare the proximal type epithelioid sarcoma should be regarded as a separate entity of particularly aggressive biologic behaviour. Its diagnosis attracts controversies and criticism related to the surgical approach and the choice of an adjuvant therapy.

**Virtual slides:**

The virtual slide(s) for this article can be found here: http://www.diagnosticpathology.diagnomx.eu/vs/1508554852942125

## Background

Among vulvar malignancies sarcomas are mainly uncommon diagnoses, in fact most of the vulvar neoplasms are represented by squamous cells carcinoma. Considering the total amount of vulvar sarcoma (1.5-5% of all vulvar neoplasia) the most common type is leiomyosarcoma, contrary the epithelioid sarcoma is so unfrequent that its diagnosis could not be reached. On the other hand if the “classical-type” epithelioid sarcoma is often indolent the “proximal-type” is a very aggressive neoplasm with a natural inclination to start spreading in the organism [1]. The dynamics of evolution of this tumor and its best treatment have not been totally defined. We report a case of vulvar “proximal-type” ephitelioid sarcoma accompanied by regional lymph node metastatic repetitions in a 63 old woman.

## Case presentation

In January 2012 a 63 year-old woman presented to the Department of Obstetrics and Gynecology of “Tor Vergata” University of Rome because of a pruritic vulvar plaque with irregular margins appeared three months before in the vulvar paraclitoral commissure area. Her family history did not reveal malignancies in first-degree relatives and her past medical history was unremarkable. She was 2 gravida 2 para and referred no previous gynecological pathologies. At gynecological examination vagina, cervix and uterus appeared normal, on the contrary a vulvar indurated and ulcerated lesion was observed (maximum diameter = 4 cm). The mass involved the clitoris and both the right and left majus and minus labium. Two excisional biopsies were taken from the primary lesion that documented a VIN3. Extended second biopsy was needed for the quickly increase of the plaque. Histological evaluation revealed nodules of neoplastic cells with the features of severe anaplasia. Immunohistochemistry the tumor was positive for vimentin, focal positivity with AE1-AE3 CK, CK5 and high molecular weight cytokeratin and negative for EMA, CD34, desmin, myosin, CroA, synaptophysin and S100. It was defined a proximal-type ephitelioid sarcoma (PES), a very infrequent kind of sarcoma. The initial diagnosis was successively confirmed by another external pathologist. Surgical margins were positive. The patient was prior submitted to a total body CT scan that did not reveal distant metastasis and a week after the diagnosis she underwent radical vulvectomy and bilateral inguinal lymph node dissection by Micheletti’s classic surgical technique [2]. The pathologist diagnosis confirmed a proximal-type ephitelioid sarcoma of the vulva.

**Figure 1:**
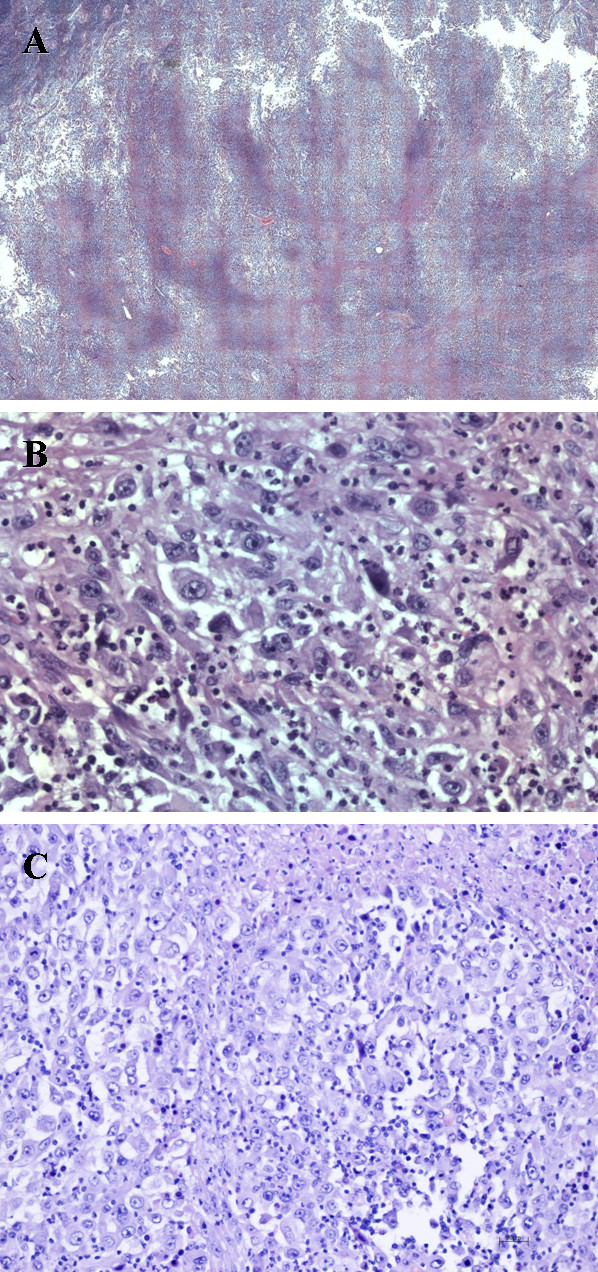
**A) Histologically the tumor was composed of nodules of polygonal epithelioid and spindle cells circumscribe areas of central hyalinization and necrosis (H&E, ×200). B)** The nodular pattern was composed by large cells with amphophilic cytoplasm. The tumor nodules were surrounded by and infiltrated by inflammatory cells. Nuclei were large and vescicolar and nucleoli were overshot (H&E, ×200). **C)** Immunostaining showed KERAE1-AE3+, VIM+, EMA-, CD34- ker 5-6-, s100-, ACTml-, MART1-, HMB45- (H&E, ×200).

Histologically the tumor was composed of nodules of polygonal epithelioid and spindle cells circumscribe areas of central hyalinization and necrosis (Figure 1A). The nodular pattern was composed by large cells with amphophilic cytoplasm. The tumor nodules were surrounded and infiltrated by inflammatory cells. Nuclei were large and vescicolar and nucleoli were overshot (Figure 1B). Immunohistochemistry the tumor was positive for vimentin, smooth muscle actin and Ki-67; focal positivity with AE1-AE3 CK, CK5 and high molecular weight cytokeratin and negative for EMA, CD34, desmin, myosin, CroA, synaptophysin and S100 (Figure 1C).

The surgical margins were free of neoplasm. One of 35 inguinal lymph nodes was metastatic. Following surgical treatment, adjuvant radiotherapy was planned. Nowadays at a 14 months-follow-up the patients is free of disease.

## Conclusions

Epithelioid sarcoma was first described by Enzinger in 1970 [3]. It is a rare soft-tissue sarcoma typically presenting as a subcutaneous or deep dermal mass and originating in distal and proximal extremities of adolescents and young adults. Typically it shows a rapid clinical evolution and tents to spread to the body in particular to the regional lymph nodes, to the lung and in the abdomen. Two different categories of epithelioid sarcoma are described in literature: in contrast to the more common “distal-type”, the “proximal-type” appears to be more aggressive even if it is very rare. Epithelioid sarcoma of the vulva was first defined by Piver et al. in 1972 [4]. It often occurs in the labia majora of young women, and frequently could be misdiagnosed as benign lesion such as infectious granuloma, Bartholin's cysts, fibroma, lipoma, dermoid cysts, fibrous histiocytoma, viral warts, or squamous cell carcinoma [5].

PES of the vulva is an extremely rare tumor, and a review of the English-language literature shows 29 cases from 1972 to 2012 [4-27], most of these papers are case reports and no extended clinical records are published by now (Table 1). Majority of the patients included in the papers were early post-menopausal women at the time of diagnosis but some of them were very young (median age 36,4 years). All of them recounted a clinical history of a tender little mass appeared in the labia majora, rapidly growing, without symptoms or only accompanied with a lasting itch. Frequently histological diagnosis was missed at first and the patients typical experience was that of repeated biopsies before definitive treatment. Literature review showed that 19 patients underwent local excision (in 8 of these cases defined as “wide excision”), 5 radical bilateral vulvectomy, 5 hemivulvectomy, in 9 cases mono or bilateral inguinal lymph node dissection was chosen.

**Table 1:** **Vulvar proximal type epithelioid sarcoma**: **the current literature review**

**Author**	**Year**	**N°**	**Age**	**Surgery**	**RT**	**CT**	**Recurrent site**	**DFS (months)**	**OS (months)**
Piver SM et al. [4]	1972	1	27	Wide local excision	No	No	None	-	NED (108)
Gallup DG et al. [6]	1976	1	31	Radical vulvectomy	No	No	None	-	NED (16)
Hall DJ et al. [7]	1980	1	31	Wide local excision, inguinal lymph node dissection	No	No	Local, lymph node, lung	35	DOD (70)
Ulbright TM et al. [8]	1983	2	55	Local excision	No	No	Local, lung, abdominal wall, skin	8	DOD (15)
			30	Wide local excision	No	Yes	Local, Lung	2	DOD (8)
Tan GWT et al. [9]	1989	1	21	Wide local excision	No	No	None	-	NED (36)
Perrone T et al. [10]	1989	1	21	Local excision, lymph node dissection	No	No	None	-	NED (56)
Wevers AC et al. [11]	1989	1	21	Local excision, lymph node dissection	No	No	Local recurrence	2	DOD (5)
Weissmann D et al. [12]	1990	1	26	Local excision	No	No	Local lymph node, lung, scalp, liver, kidney	14	AWD (78)
Hernandez-Ortiz MJ et al. [13]	1995	1	51	Radical vulvectomy	No	No	Lung	5	DOD (8)
Konefka T et al. [14]	1994	1	49	Radical vulvectomy and bilateral inguinal lymph node dissection	No	Yes	Lung	5	DOD (8)
Guillou L et al. [15]	1997	3	57	Local excision	Yes	No	None	-	DOID (52)
			49	Radical vulvectomy	Yes	No	None	-	NED (22)
			45	Wide local excision	No	Yes	None	-	NED (21)
Tjalma WA et al. [16]	1999	1	23	Hemivulvectomy	No	No	None	-	NED (48)
Kasamatsu T et al. [17]	2001	1	23	Local excision	No	No	Local lymph node	96	NED
	2001	3	37	Local excision	No	Yes	None	-	NED (11)
			30	Local excision	No	No	Local lymph node, skin	96	NED (104)
			80	Wide local excision	No	No	Local lymph node, lung	16	DOD (23)
Altundag K et al. [5]	2004	1	51	Left hemivulvectomy with bilateral inguinal lymph node dissection	No	Yes	Lung	4	DOD (6)
Dainese E et al. [19]	2005	1	34	Right emivulvectomy with right inguinal lymph node dissection	No	No	None	-	NED (12)
Argenta PA et al. [20]	2007	1	35	Radical excision, inguinal-femoral lymph node dissection	Yes	No	None	-	NED (40)
Kim JH et al. [21]	2008	1	24	Clitoris sparing wide local excision	No	No	None	-	NED (8)
Rai H et al. [22]	2009	1	17	Local excision, lymph node dissection	Yes	No	Local lymphnode	2	AWD (2)
Tholpady A et al. [23]	2010	1	17	Radical excision	No	No	-	-	NED (12)
Chiyoda T et al. [24]	2011	1	33	Left radical vulvectomy	No	No	None	-	NED (36)
Andrisani A et al. [25]	2011	1	46	Radical superior vulvectomy	Yes	Yes	Local lymph nodes, abdominal wall, genital, inguinal and thigh muscles, bladder	1	DOD (11)
Kim HJ et al. [26]	2012	1	41	Local excision	No	No	None	-	NED (10)
Ong AC et al. [27]	2012	1	51	Right emivulvectomy and right inguinal lymph node dissection	No	No	None	-	NED (8)
Our study		1	63	Bilateral radical vulvectomy, right inguinal lymph node dissection	Yes	No	Inguinal lymph node	-	NED (14)

*NED* no evidence of disease, *DOD* dead of disease, *DOID* dead of inter current disease, *AWD* alive with disease, *RT* radiotherapy, *CT* chemotherapy, *DFS* disease free survival, *OS* overall survival.

Among the group submitted to radical surgery (5 mono e 5 bilateral vulvectomy) median over all survival (OS) was 17.7 months (range 8–48 months), median disease free survival (DFS) was 15.7 months (range 1–48 months), 4 patients died of disease (DOD) with lung, local lymph nodes, abdominal wall, genital, inguinal, thigh muscles and bladder metastasis within a median of 8 months after surgery (range 6–11 months).

Among the group submitted to local excision (19 patients) median OS was 39.7 months (range 2–108 months), median DFS was 32.8 months (range 2–96 months). Five patients DOD within a median of 24.2 months after surgery (range 5–70 months), a patient was alive with disease (AWD) 78 months after surgery and a patient died of inter current disease 52 months after surgery.

Ten patients were submitted to inguinal lymph node dissection at the time of the first surgery (5 together with radical surgery and 5 with local excision), 5 DOD after a median of 19,4 months (range 5–56 months), two are alive with no evidence of disease after a median of 19 months (range 12–56 months) and a patient is AWD at a 2 months follow-up.

Only for 10 patients of the entire review adjuvant therapy was indicated, 5 of them received radiotherapy and 6 chemiotherapy. Among this group 4 patients DOD (median OS 8.25 months, median DFS 3 months), a patient died of inter current disease after 52 months, 4 patients are alive with no evidence of disease (median OS 23.5 months) and a patient is alive with local lymph node metastasis after 2 months follow-up. As concerned the tendency to spread lung, skin and local lymph node were preferred target sites for metastasis. 12 patients have a metastatic disease and the median time to relapse was 23,6 months after surgery (range 1–96 months).

From the data reported in the literature there is no treatment of choice universally approved for vulvar epithelioid sarcoma. Approximately 70-77% of patients have local recurrence [8]. That’s why immediately local excision is considered mandatory. Local wide excision or radical vulvectomy is usually performed including 2 cm from the margin of tumor. There is no evidence of the beneficial effect of lymph node resection on local or distant relapse rate and inguinal lymph node dissection should be consider only when they are clinically suspicious or enlarged. The roles of adjuvant radiotherapy and chemotherapy are not proved. Any benefit of radiotherapy and/or chemotherapy in preventing recurrence or for palliation has not been demonstrated. When surgery and radiotherapy are combined a local better control can be achieved but 40-60% of patients with high-grade sarcomas will die of metastatic disease. For this reason the early detection of disease is crucial. Ulutin et al. [1] and DiSaia et al. [28] reported a median time of 6 months for the diagnosis of vulvar sarcoma. A classic clinical behaviour consists in a slowly growing painless mass in labia majora. The lack of symptoms in the first period and the rapid tendency of spread to the body justify the poor prognosis of these cases.

Given the varied morphologic features and immunophenotypic heterogeneity of the present case, PES can potentially be mistaken for other epithelioid malignancy. Immunohistochemical studies on PES show immunoreactivity for vimentin, cytokeratin, and ephitelial membrane antingen (MEA). S 100 and CD31 are negative, while desmin and CD34 are positive in some cases [18]. Langerhans cell sarcoma (LCS) or mammary sarcoma (MS) may be similar to PES but immunohistochemistry, in the LCS the tumour cells are positive for CD1a, S-100 protein and langerin strongly and diffusely [29], while in MS CD10 and vimentin are positive diffusely whereas epithelial markers and other myoepithelial or myogenic markers were all negative [30]. In Prostatic stromal sarcoma (PSS) unlike the PES, immunohistochemically, the tumour cells are widely positive for vimentin, CD56, CD99 and focally positive for synaptophysin, CD10, progesterone receptor, desmin and CD34, but negative for EMA, cytokeratin, estrogen receptor, S-100 and myoglobin [31]. Epithelioid malignant peripheral nerve sheath tumors (MPNSTs) might be positive for cytokeratin and EMA occasionally, but about 80% of cases of MPNST show diffuse and strong reactivity for S-100 protein [32]. Similarly, malignant melanomas are typically strongly immunoreactive for S-100 protein and gp100 protein (HMB-45). A particular differential diagnosis between proximal epithelioid sarcoma and malignant rhabdoid tumor (MRT) could be arduous but of extremely importance. The distinguishing between them could be reached using an innovative molecular marker, INI1, as suggested by some authors. In fact the tumors from the two different categories lack the INI1 gene product. Nevertheless, the literature suggests that other selected genetic differences between the 2 lesions, and the more rapid and aggressive course of MRT distinguish these tumor types as separate clinicopathologic entities [26]. Even the epithelioid angiosarcoma may be similar to the PES, in fact immunohistochemically the tumor is positive for the pan-cytokeratin, p63, cytokeratin18, Vimentin and vascular markers CD31, and is negative for CD34, cytokeratin5/6, cytokeratin7, cytokeratin20, CD68, CD30, S-100, HMB45, desmin, α-SMA and CD45 [33].

Epithelioid sarcomas are easily distinguished from leiomyosarcomas by their lacking a representative area of a leiomyosarcoma, showing fascicles of elongated tumor cells with blunt-ended "cigar-shaped" atypical nuclei, and greater frequency of negativity for desmin and SMA [34].

The distinction between proximal-type epithelioid sarcoma and undifferentiated carcinoma is probably the most difficult consideration [35]. The occurrence of tumors in the subcutis or deep soft tissues without any connection with the overlying epidermis or cutaneous adnexa, the absence of histologic features of squamous or glandular differentiation, and presence of CD34 reactivity in about half of the cases favor the diagnosis of epithelioid sarcoma over undifferentiated carcinoma. The latter are negative for CD34 in most cases [36].

In conclusion the PES of the vulva is rare, radical surgery with lymphadenectomy and adjuvant therapy seems not to guarantee better DFS and OS and early detection plays a crucial role. For this reason these cases should be directed to referral centers of oncology.

## Consent

Written informed consent was obtained by patient for publication of this report and any accompanying images. A copy of the written consent is available for review by the Editor-in-Chief of this journal.

## Competing interests

The authors declare that they have no competing interest.

## Authors’ contributions

All the authors contributed to the acquisition of data, revised the paper and gave final approval.
